# Transcriptional profiling of circulating extracellular vesicles from prebiopsy prostate cancer patients

**DOI:** 10.1002/1878-0261.70244

**Published:** 2026-03-26

**Authors:** Stefan Werner, Pierre Tennstedt, Randi C. Pose, Christian Müller, Katharina Besler, Svenja Schneegans, Malik Alawi, Marie C. Roesch, Sven Peine, Desiree Bonci, Joanna Budna‐Tukan, Evi Lianidou, Catherine Alix‐Panabières, Derya Tilki, Klaus Pantel

**Affiliations:** ^1^ Department of Tumor Biology University Medical Center Hamburg‐Eppendorf Germany; ^2^ The PROLIPSY consortium Hamburg Germany; ^3^ Martini‐Klinik Prostate Cancer Center, University Hospital Hamburg‐Eppendorf Germany; ^4^ Bioinformatics Core, University Medical Center Hamburg‐Eppendorf Germany; ^5^ Department of Urology University Hospital Schleswig‐Holstein, Campus Lübeck Germany; ^6^ Department of Transfusion Medicine University Medical Center Hamburg‐Eppendorf Germany; ^7^ Istituto Superiore di Sanità Rome Italy; ^8^ Department of Immunology Poznan University of Medical Sciences Poznan Poland; ^9^ Department of Histology and Embryology Poznan University of Medical Sciences Poznan Poland; ^10^ Department of Anatomy and Histology, Collegium Medicum University of Zielona Gora Poland; ^11^ Department of Analytical Chemistry University of Athens Greece; ^12^ Laboratory of Rare Human Circulating Cells, University Medical Centre of Montpellier IURC France

**Keywords:** early detection, gene expression analysis, liquid biopsy, prostate cancer, transcriptional profiling, tumor‐derived extracellular vesicles

## Abstract

Early detection of cancer is essential for effective treatment. However, current prostate cancer screening methods lack sufficient sensitivity and specificity, leading to overdiagnosis and unnecessary treatment. There is also an unmet need to distinguish clinically significant from insignificant prostate cancer. To identify complementary biomarkers for improved screening and diagnosis, we performed transcriptional profiling of cancer‐associated transcripts in circulating extracellular vesicles (EVs) isolated from peripheral blood of patients with suspected prostate cancer prior to biopsy and healthy donors. Expression data for 2549 mRNAs were obtained from 28 men. CAPN5 expression was significantly lower, whereas *BIRC2, CASP3, CD63, FMO5, IRF6, PFDN1, PRDX6, PSMD2, RIT1, S100A2, THBS1*, and *XRCC2* were significantly elevated in EVs from patients with significant prostate cancer (*n* = 14) compared with cancer‐free individuals and patients with insignificant disease (*n* = 14). Candidate biomarkers were subsequently evaluated by *in silico* validation using the The Cancer Genome Atlas (TCGA) prostate adenocarcinoma dataset and the GEO dataset GSE70768 containing benign and malignant prostate tissues. This analysis identified *CASP3*, *XRCC2*, and *RIT1* transcripts in circulating EVs as promising biomarkers for the early detection of significant prostate cancer.

## Graphical abstract

RNA profiling of circulating extracellular vesicles (EVs) from blood samples of men undergoing prostate biopsy identifies transcripts associated with clinically significant prostate cancer. Integrative analysis with public tumor datasets links EV‐derived gene signatures to tumor stage and progression‐free survival, highlighting CASP3, XRCC2, and RIT1 as candidate biomarkers for early detection. Created with ChatGPT (OpenAI, GPT‐5.4 Thinking) based on a user prompt.

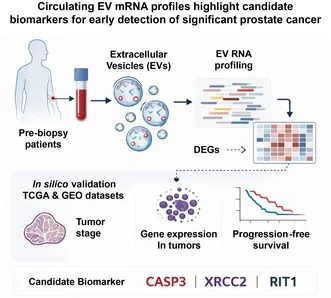

**Abbreviations**
BIRC2: Gene, baculoviral IAP repeat‐containing protein 2
CAPN5: Gene, calpain‐5
CASP3: Gene, caspase 3
CD63: Gene, cluster of differentiation 63
EV: Extracellular vesicles
FDR: False discovery rate
FMO5: Gene, flavin‐containing monooxygenase 5
IRF6: Gene, interferon regulatory factor 6
PC: Prostate Cancer
PFDN1: Gene, prefoldin Subunit 1
PRDX6: Gene, peroxiredoxin 6
PSA: Prostate‐specific antigen
PSMD2: Gene, 26S proteasome non‐ATPase regulatory subunit 2
RIT1: Gene, ras‐like without CAAX 1
S100A2: Gene, S100 Calcium Binding Protein A2
TCGA: The cancer genome atlas
THBS1: Gene, thrombospondin 1
XRCC2: Gene, x‐ray repair cross complementing

## Introduction

Prostate cancer (PC) is a leading cause of cancer‐related death in men [1]. To reduce cancer‐related death, prostate‐specific antigen (PSA) screening is the preferred blood test. However, PSA screening leads to considerable overdiagnosis and subsequent overtreatment. Further, PSA is not suitable to discriminate between clinically insignificant from significant PC [2]. Reducing the overdiagnosis of PC caused by current clinical practice is therefore a major unmet need.

The course of PC is highly variable from patient to patient, ranging from indolent disease to highly aggressive and lethal disease. Defining clinically significant prostate cancer is also an important concern in prognosis to inform patient management and clinical decisions. The lack of reliable tests to identify tumors destined to remain insignificant has led to significant overtreatment of patients with no life‐threatening disease [3]. For the decisive detection and diagnosis of PC, a prostate needle biopsy is regularly performed. Ultrasound guided prostate biopsy has an overall detection rate of about 50% of tumor cases. As an invasive procedure, this can be associated with pain and in some cases with complications such as infections [4]. Therefore, it may be beneficial to complement existing procedures for early detection and refinement of PC diagnosis with less invasive methods that provide molecular information.

The analysis of body fluids like peripheral blood for tumor cells or tumor‐derived material such as nucleic acids is known as ‘liquid biopsy’. This concept has opened new avenues in cancer diagnostics, including early detection of tumors, improved risk assessment and staging, as well as early detection of relapse and monitoring of tumor progression in the context of cancer therapies [5]. Extracellular vesicles (EVs) are membrane‐bound particles of various sizes that can carry diverse functional molecular cargoes including proteins, lipids, DNA, messenger, and noncoding RNAs to promote intercellular communication and may be involved in cancer development [6]. The expression of miRNAs in EVs isolated from the blood, semen, and urine of PC patients is a promising biomarker [7, 8, 9]. Changes in the expression of selected protein‐coding messenger RNAs (mRNAs) contained in EVs isolated from PC patients have already been analyzed by mRNA‐sequencing or PCR‐based approaches to establish a potential prognosis biomarker [10] or to detect therapy resistance [11]. In advanced prostate cancer, several studies have demonstrated that RNA derived from extracellular vesicles can be reliably detected in patient samples and provide clinically meaningful information. EV RNA analyses have been shown to reflect tumor biology, predict treatment resistance, monitor disease progression, and assess patient outcome, supporting the feasibility and clinical relevance of EV RNA‐based liquid biopsy approaches in this clinical setting [12, 13, 14]. Urine‐based tests are already used to help decide whether men with suspected prostate cancer should undergo prostate biopsy. These tests analyze RNAs carried by EVs and improve the detection of clinically significant cancer compared with PSA alone, while reducing unnecessary biopsies [15, 16, 17]. However, the content of mRNAs in EVs isolated from the blood of early‐stage PC patients has not been investigated in detail. So far, little is known about the connection between the content of protein‐coding mRNA in EVs and the risk of developing prostate cancer [18].

In this study, we sought to characterize the mRNA content of EVs isolated from the blood of suspected PC patients and age‐matched healthy individuals. By comparing the mRNA profiles in groups of healthy individuals and patients with insignificant to patients with significant PC, we aim to identify transcripts as potential biomarkers for early detection of significant PC.

## Materials and methods

### Blood collection

Informed consent was obtained from all participants prior to enrolment. The study was conducted in accordance with the ethical principles of the Declaration of Helsinki (World Medical Association, 2013) and was approved by the ethical commission of the Hamburger Ärztekammer (reference number PV5392). Before biopsy, 77 blood samples were taken from patients with suspected prostate cancer at the Martini‐Klinik at the University Medical Center Hamburg‐Eppendorf. Samples were collected between January 2019 and December 2021. The experiments were undertaken with the understanding and written consent of each subject. The inclusion criteria were defined as men of full age with suspected prostate cancer and a PSA level of at least 4 ng/mL, who had been designated for biopsy. The exclusion criteria were verified viral infections (HIV or hepatitis), finasteride treatment, and other diagnosed malignancies. The blood (7.5 mL) was drawn into EDTA‐blood collection tubes and processed within 3 h. Blood samples from 12 age‐matched healthy male blood donors were obtained as negative control from the Institute of Transfusion Medicine, Medical Center Hamburg‐Eppendorf. The summary of patients' characteristics is provided in Table S1.

### Plasma, EV and RNA isolation

For plasma isolation, peripheral blood samples were centrifuged at 1500 g for 10 min at room temperature. Plasma was collected with a Pasteur pipette, mixed, transferred to cryovials, and stored at −80 °C until EV isolation. For EV isolation, the sortev RNA Kit (EXOSOMICS Spa, Siena, Italy) was used according to the manufacturer's instructions. The kit employs an immunocapture‐based strategy to enrich tumor‐derived EVs. Its provider holds a patent related to the selective enrichment of carbonic anhydrase IX (CA‐IX)‐positive EVs [19]. Previous studies have demonstrated the kit's and method's efficacy in isolating EVs from prostate and other cancer cells [20, 21, 22].

### Transcriptional profiling

For transcriptional profiling, we used the HTG EdgeSeq service with the Oncology Biomarker Panel, which consists of 2549 preselected genes associated with cancer biology. A total of 89 samples were sequenced, but only 28 samples passed the HTG EdgeSeq postsequencing metrics and were subjected to differential gene expression analysis. About 16 SoRTEV‐isolated human plasma EV samples were omitted due to poor sample quality, measured by percentage of mapping reads to given position along transcripts (POS%) with a threshold of > 15% failure. About 45 samples were successfully sequenced but had a very low standard deviation between individual gene expression levels and therefore did not meet the minimum expression viability. These samples were not considered informative and were excluded from the bioinformatic analysis.

For the remaining 28 samples, expression residuals were calculated using the negative binomial generalized linear model functions from the R/Bioconductor edgeR package after upper‐quartile normalization [23]. Based on the residuals, one hidden factor of unwanted variation was calculated by the RUVr method from the package RUVSeq [24] and used as a covariate in the differential expression analysis based on DESeq2 [25]. A gene was considered differentially expressed if the corresponding false discovery rate (FDR) ≤ 0.1.

### *In silico* validation

For *in silico* validation, preprocessed gene expression data normalized to normal tissue together with corresponding clinical information from the TCGA prostate adenocarcinoma cohort [26] comprising 494 prostate cancer patients were retrieved from the cBio Cancer Genomics Portal [27]. To assess associations between candidate gene expression and tumor progression, gene expression levels were analyzed across tumor stage groups defined according to the American Joint Committee on Cancer (AJCC) tumor stage classification. To evaluate the relationship between gene expression and clinical outcome, progression‐free survival (PFS) analyses were performed by stratifying patients according to gene expression levels. To compare gene expression levels between benign and malignant prostate tissues, preprocessed expression data from the Gene Expression Omnibus dataset [28] were analyzed.

### Statistical analysis

Statistical analyses were performed using R software and graphpad Prism. Differences in EV transcript expression between the no cancer/low‐risk group and patients with significant prostate cancer were assessed using a two‐tailed Student's *t*‐test. For the TCGA validation cohort, differences in gene expression across tumor stage groups were evaluated using one‐way analysis of variance (anova). Progression‐free survival (PFS) was analyzed using Kaplan–Meier survival curves, and differences between groups were assessed using the log‐rank test. Comparisons of gene expression between benign and malignant prostate tissues in the GSE70768 dataset were performed using an unpaired two‐tailed Student's *t*‐test. All statistical tests were two‐sided, and *P* values < 0.05 were considered statistically significant.

## Results

### Identification of differentially expressed EV transcripts associated with significant prostate cancer

To identify differentially expressed genes in EVs from PC patients and noncancer individuals, we collected blood samples from men with suspected PC just prior to prostate biopsy. Based on the pathological assessment of their respective biopsies, these patients were classified as having no cancer, low‐risk PC with a Gleason score of 6, and significant PC with a Gleason score greater than 6. As an additional control, we took blood samples from age‐matched individuals who were blood donors. These individuals were considered to be cancer‐free healthy controls. EVs from 7.5 mL of blood from 89 individuals were isolated by antibody capture and processed for mRNA sequencing. Due to poor RNA quality or lack of overall expression viability, 61 samples were not considered informative and were excluded from bioinformatic analysis. About 28 samples passed HTG EdgeSeq postsequencing metrics and were considered informative and subjected to gene expression analysis. We first performed a differential gene expression analysis in groups of healthy controls, noncancer patients and low‐risk PC patients with a Gleason score of 6 (no cancer/low‐risk) versus all patients with significant prostate cancer (significant cancer) with a Gleason score greater than 6. The gene expression of *CAPN5* was found to be significantly lower in EVs isolated from no cancer/low‐risk individuals. However, the expression of *BIRC2, CASP3, CD63, FMO5, IRF6, PFDN1, PRDX6, PSMD2, RIT1, S100A2, THBS1*, and *XRCC2* was significantly higher in EVs isolated from blood samples of patients with significant cancer compared to cancer‐free individuals (Fig. 1A,B). Thus, the detection of these transcripts in EVs isolated from peripheral blood samples represents candidate biomarkers for the detection of patients with significant PC. Using unsupervised hierarchical clustering analysis, we found that only one sample from the significant cancer group clustered together with the no cancer/low risk samples (Fig. 1C).

**Fig. 1:**
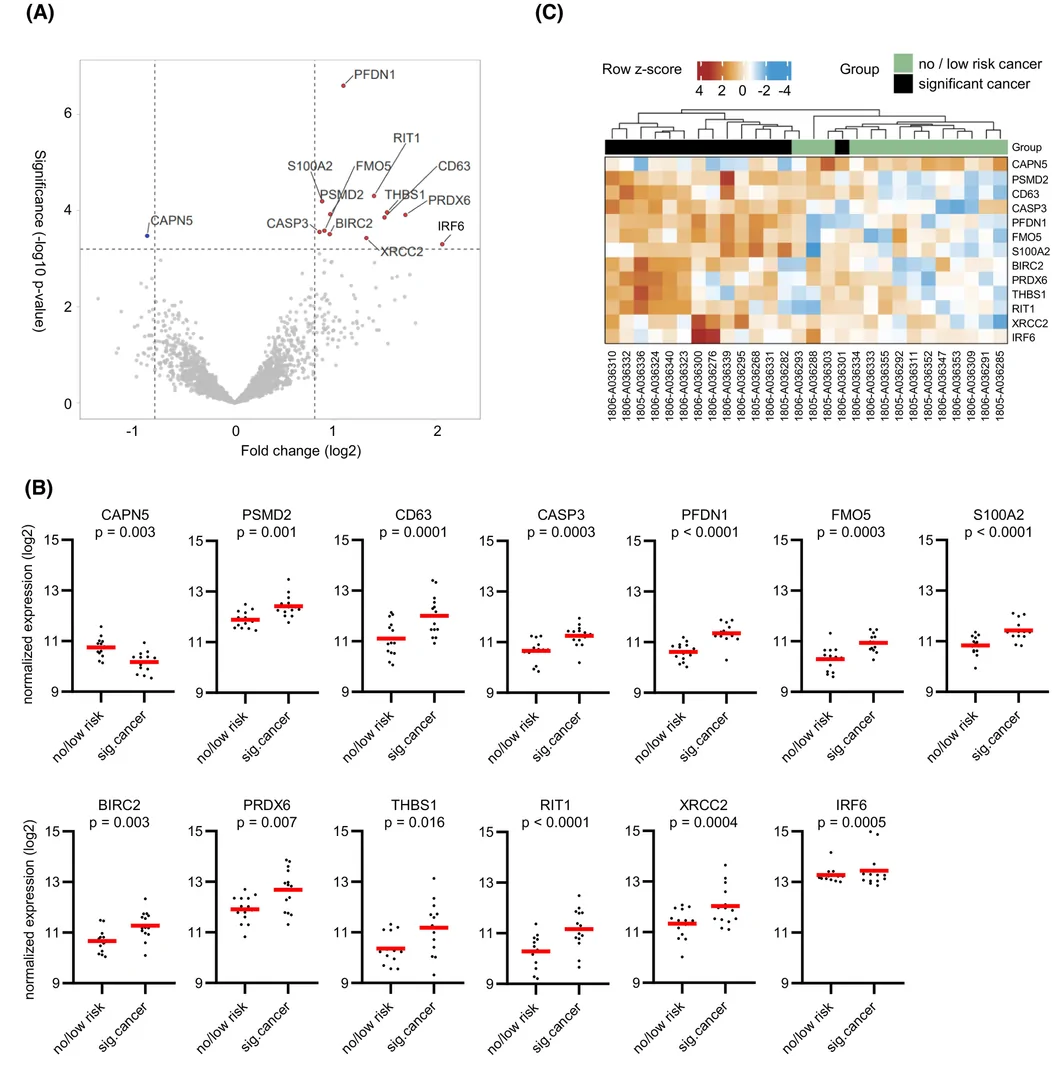
Analysis of the mRNA content of circulating extracellular vesicles (EVs) for differential gene expression. (A) Volcano plot summarizing differential gene expression in EVs isolated from plasma of patients with significant prostate cancer (‘significant cancer’) compared with healthy donors, noncancer individuals, and patients with low‐risk prostate cancer (‘no cancer/low risk’). Genes marked in red are significantly upregulated in the significant cancer group. Differential expression was defined as |log_2_ fold change (log_2_FC)| ≥ 1 with a false discovery rate (FDR) ≤ 0.1. (B) Direct comparison of the relative expression levels of the indicated genes between the no cancer/low risk and significant cancer groups. Each dot represents one patient sample. The red horizontal line indicates the mean expression level of each group. EV RNA expression was measured once per sample without technical replicates. Statistical comparisons between groups were performed using a two‐tailed Student's *t*‐test. Sample sizes were as follows: significant cancer (*n* = 14) and no cancer/low risk (*n* = 14). (C) Heat map showing unsupervised hierarchical clustering of samples based on EV gene expression profiles. The heat map includes all genes that were differentially expressed (|log_2_FC| ≥ 1 and FDR ≤ 0.1). Expression values are shown as row z‐scores of normalized log_2_ expression values.

### *In silico* validation associates candidate EV transcripts with tumor stage, progression‐free survival, and differential expression in prostate cancer tissue

To validate the clinical relevance of the identified transcripts in PC progression, we examined the TCGA dataset of prostate adenocarcinomas [27] we examined the TCGA dataset of prostate adenocarcinomas We confirmed that the expression of the *CASP3*, *XRCC2*, *RIT1*, *PSMD2*, and *IRF6* genes in primary tumors increased steadily with higher tumor stages (Fig. 2A, Fig. S1). However, there were no significant differences in the expression of *PFDN1*, *PRDX6*, *BIRC2*, *THBS1*, *S100A2*, and *CD63* across tumor stages. Contrary to the liquid biopsy results, we observed significantly decreased *FMO5* gene expression and significantly increased *CAPN5* gene expression in advanced tumor stages (Fig. S1).

**Fig. 2:**
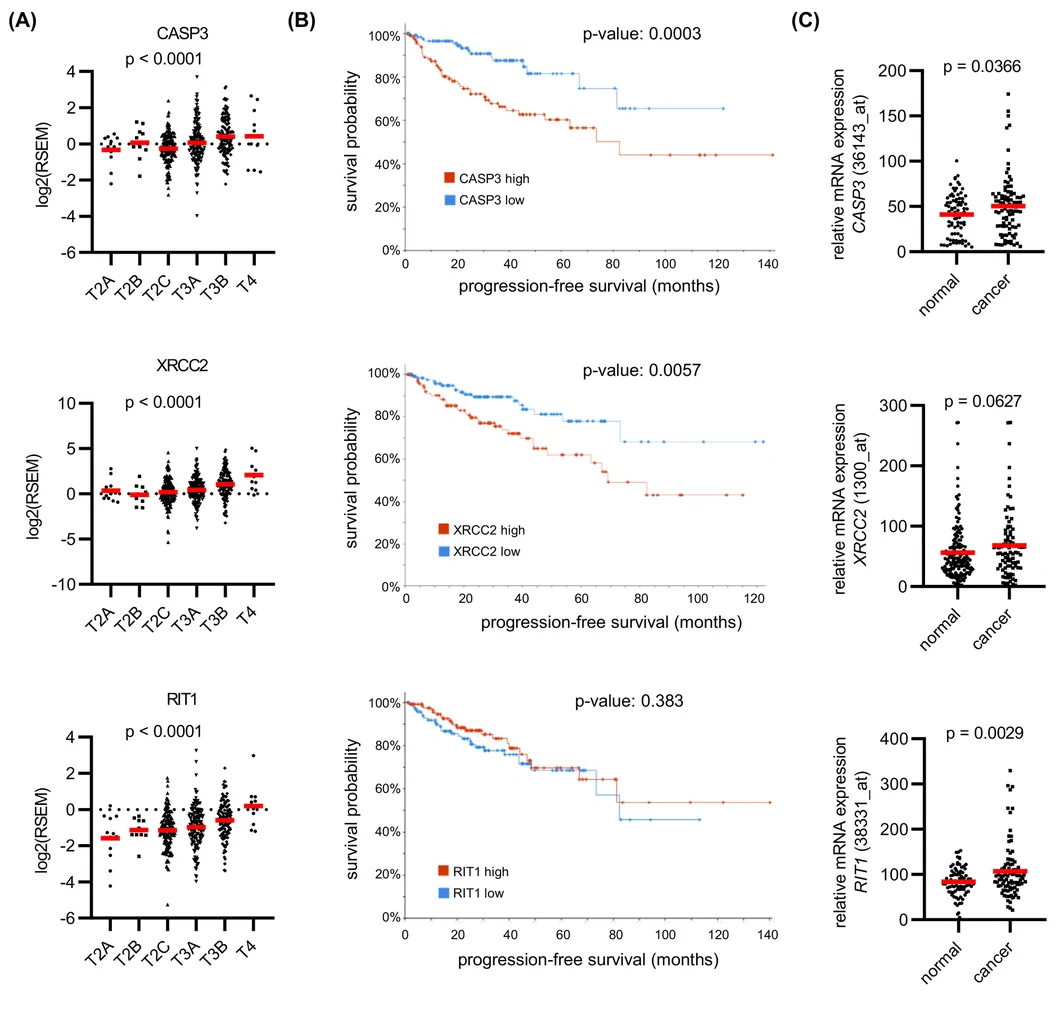
*In silico* validation of *CASP3*, *XRCC2*, and *RIT1* in published gene expression datasets of prostate cancer. (A) Stage‐dependent expression analysis of *CASP3*, *XRCC2*, and *RIT1* using RNA‐sequencing data from the TCGA PanCancer prostate adenocarcinoma dataset. Gene expression values are shown as log_2_‐transformed RSEM‐normalized counts across tumor stages defined according to the AJCC tumor staging system. Sample sizes were as follows: T2A (*n* = 13), T2B (*n* = 10), T2C (*n* = 163), T3A (*n* = 157), T3B (*n* = 133), and T4 (*n* = 10). Each dot represents an individual patient sample, and the red horizontal line indicates the median expression level for each stage group. Differences in gene expression across tumor stages were evaluated using the Kruskal–Wallis nonparametric test, which assesses whether expression distributions differ across all tumor stage groups simultaneously. The *p*‐values shown in each panel correspond to this global comparison across the tumor stage groups. (B) Kaplan–Meier analysis of progression‐free survival (PFS) in prostate adenocarcinoma patients from the TCGA PanCancer dataset according to expression levels of *CASP3*, *XRCC2*, and *RIT1*. Patients were stratified by gene expression, and the highest expression quartile (top 25%) was compared with the lowest expression quartile (bottom 25%). Kaplan–Meier curves represent the probability of progression‐free survival over time (months) for each group. Survival differences between the high‐ and low‐expression groups were evaluated using the log‐rank test. The p‐values shown in the panels correspond to this comparison between high‐ and low‐expression patient groups. (C) Independent validation of gene expression differences between normal prostate tissue and prostate cancer samples using microarray data from the GSE70768 dataset (Gene Expression Omnibus). Relative mRNA expression levels for *CASP3*, *XRCC2*, and *RIT1* are shown for normal prostate tissue samples and prostate cancer samples. Each dot represents an individual patient sample, and the red horizontal line indicates the mean expression level for each group. Differences between normal and cancer samples were evaluated using an unpaired two‐tailed Student's *t*‐test, and the *p*‐values shown correspond to the comparison between the two groups. Sample sizes were: normal (*n* = 81) and cancer (*n* = 90).

We validated the correlation between high *CASP3*, *XRCC2*, and *PFDN1* gene expression and progression‐free survival (Fig. 2B and Fig. S2). No significant associations were found between progression‐free survival and the expression levels of *RIT1*, *PSMD2*, *IRF6*, *PRDX6*, *BIRC2*, *THBS1*, *S100A2*, *CD63*, or *CAPN5*. We found that low *FMO5* expression was associated with shorter progression‐free survival (see Fig. S2).

To analyze whether there is an increased expression of the candidate transcripts in cancer tissue compared to benign tissue, we also used another gene expression dataset of 482 samples of benign and tumor prostate tissue [28]. We found that the expression of *CASP3* and *RIT1* was increased in tumor tissue compared with benign prostate tissue, while *XRCC2* showed borderline statistical significance (Fig. 2C). For *PSMD2*, *IRF6*, *PRDX6*, *BIRC2, THBS1, FMO5*, and *CAPN5*, no significant associations were found (Fig. S3). In contrast to the liquid biopsy results, analysis of *PFDN1*, *S100A2*, and *CD63* expression revealed inconsistent associations, as they were downregulated in tumor tissue (see Fig. S3).

In conclusion, we found that among the identified transcripts, particularly *CASP3, XRCC2, RIT1* transcripts isolated from circulating EVs are candidate biomarkers for detection of significant PC, because in primary PC their expression steadily increases with tumor stage, correlates with progression‐free survival and/or is increased in cancer tissue compared to baseline prostate tissue.

## Discussion

Several blood‐based biomarkers have been developed for early detection of PC [18, 29]. To our best knowledge, this is the first study comprehensively analyzing gene expression with a panel of 2549 cancer‐associated transcripts in circulating EVs. Due to the large number of parallel analyses of gene expression in a relatively small sample size, there is a high risk that significant results are due to chance rather than relevant biological events. We found a significant increase in mRNA levels of *CASP3*, *XRCC2*, *RIT1* in EVs of patients with significant PC and validated in primary PC tumors that their increased expression is correlated with disease progression.

The Caspase 3 protein encoded by the *CASP3* gene plays a central role in the execution‐phase of cell apoptosis [30]. It is considered that the apoptotic program of a cell can be triggered by oncogene activation and that the elimination of cells carrying activated oncogenes by apoptosis may be the primary means by which such mutant cells are continuously removed from the body's tissues [31]. However, conflicting results are published showing that decreased CASP3 protein is a prognostic marker in advanced tumor stages [32, 33]. The *XRCC2* codes for the X‐Ray Repair Cross Complementing 2 protein, which functions in homologous recombination to maintain chromosome stability and repair DNA damage [34]. Thus, the increased expression of *XRCC2* measured here may be a surrogate for the emergence of genomic instability as an indicator of PC formation, which is also reflected in our reanalysis of the TCGA dataset. As an active apoptotic program and competent DNA repair would normally eliminate abnormal cells before cancer develops, we hypothesize that elevated CASP3 and XRCC2 mRNA levels in plasma‐derived EVs may indicate a compensatory response to ongoing cellular stress, apoptosis, and genomic instability in proliferating tumor cells. This is consistent with the idea that the apoptotic and DNA repair machinery is activated but ultimately insufficient in growing tumors.

The *RIT1* gene, which encodes a member of a subfamily of Ras‐related GTPases and mediates resistance to oxidative stress [35], has not yet been described to have a role in prostate progression. However, a distinct expression and mutational profile of *RIT1* exist in diverse cancers and is not strictly limited to specific tissues [36], while elevated expression of *RIT1* correlates with poor prognosis in endometrial cancer [37].

In this study, we successfully analyzed the mRNA content of EVs in 28 samples, so our analysis was performed with relatively small sample groups and reduced statistical power. In any case, a validation study in an independent sample set is mandatory. Our analysis was hampered by a high frequency of attrition of samples that were successfully sequenced but did not show a difference in expression of the genes studied and were therefore considered noninformative. We started with 89 samples but removed 16 from the analysis due to poor mRNA quality. Compared to other nucleic acids used as liquid biopsy analytes, such as circulating DNA or microRNA, mRNA is known to be the least stable [38]. Although all blood samples collected were processed within three hours of blood collection, to reduce the incidence of dropout due to poor mRNA quality in future research, unnecessary incubation time should be eliminated, and the workflow should be streamlined toward direct sample processing and RNA sequencing immediately after blood collection. Furthermore, this study did not involve EV characterization, which introduces potential uncertainty regarding the molecular features of the enriched particles and the presence of platelets. However, most of the samples (45) that were excluded had good mRNA quality but did not pass the postsequencing RNA‐sequencing metrics because the analyzed genes in these samples appeared to exhibit similar expression levels. This suggests that quality issues are not the primary explanation. As defined by the service provider, most of the dropouts were due to minimal expression variability, of which is determined by the relative standard deviation of reads allocated to each probe within a sample. Thus, although the sequencing has been technically successful, no relevant information could be extracted. Therefore, as a reasonable follow‐up study, we propose to analyze and validate the identified individual mRNA biomarkers using PCR‐based methods either in isolated EVs as in our study or directly from plasma as done by another research group [39]. Another limitation of this study is the lack of functional validation in preclinical models. Therefore, we cannot provide evidence that the identified upregulated transcripts are drivers of tumor progression.

At the time of blood sampling, all patients in the no cancer/low‐risk group did not have a pathologic diagnosis of significant PC. However, it is possible that some of these men tested positive for PC at a later date, which could have undetermined effects on the result of this RNA‐sequencing analysis.

## Conclusions

The detection of increased *CASP3*, *XRCC2*, and *PFDN1* gene expression in circulating EVs is a candidate biomarker for significant PC. A validation study on an independent set of samples is mandatory to prove the utility of these biomarkers.

## Conflict of interest

The authors declare no conflict of interest.

## Author contributions

Conceptualization: SW, PT, MA, DT, and KP. Investigations: KB and SS. Formal analysis: SW, PT, MA, and CM. Resources: RP, MCR, and SP. Funding acquisition: DB, JB, EL, CAP, and KP. Writing: SW. Editing: KP and DB.

## Supporting information


**Fig. S1.** Expression of candidate biomarker transcripts across prostate cancer tumor stages in the TCGA PanCancer cohort.

**Fig. S2.** Association between candidate biomarker transcript expression and progression‐free survival in prostate cancer.

**Fig. S3.** *In silico* validation of candidate biomarker transcripts in in dataset GSE70768 (Gene Expression Omnibus).

**Table S1.** Sample and patient characteristics.

## Data Availability

Microarray and sequence data have been submitted to the European Nucleotide Archive (ENA); they are publicly available under accession PRJEB80122 and PRJEB80907, respectively.
