# Supplementary material for: Transcriptional profiling of circulating extracellular vesicles from prebiopsy prostate cancer patients
Source: Mol Oncol. 2026 Mar 26;20(11):2647–55. doi: 10.1002/1878-0261.70244 (PMC13398947; doi:10.1002/1878-0261.70244)
Supplement: Supplementary file 1 [file MOL2-20-2647-s002.pdf]

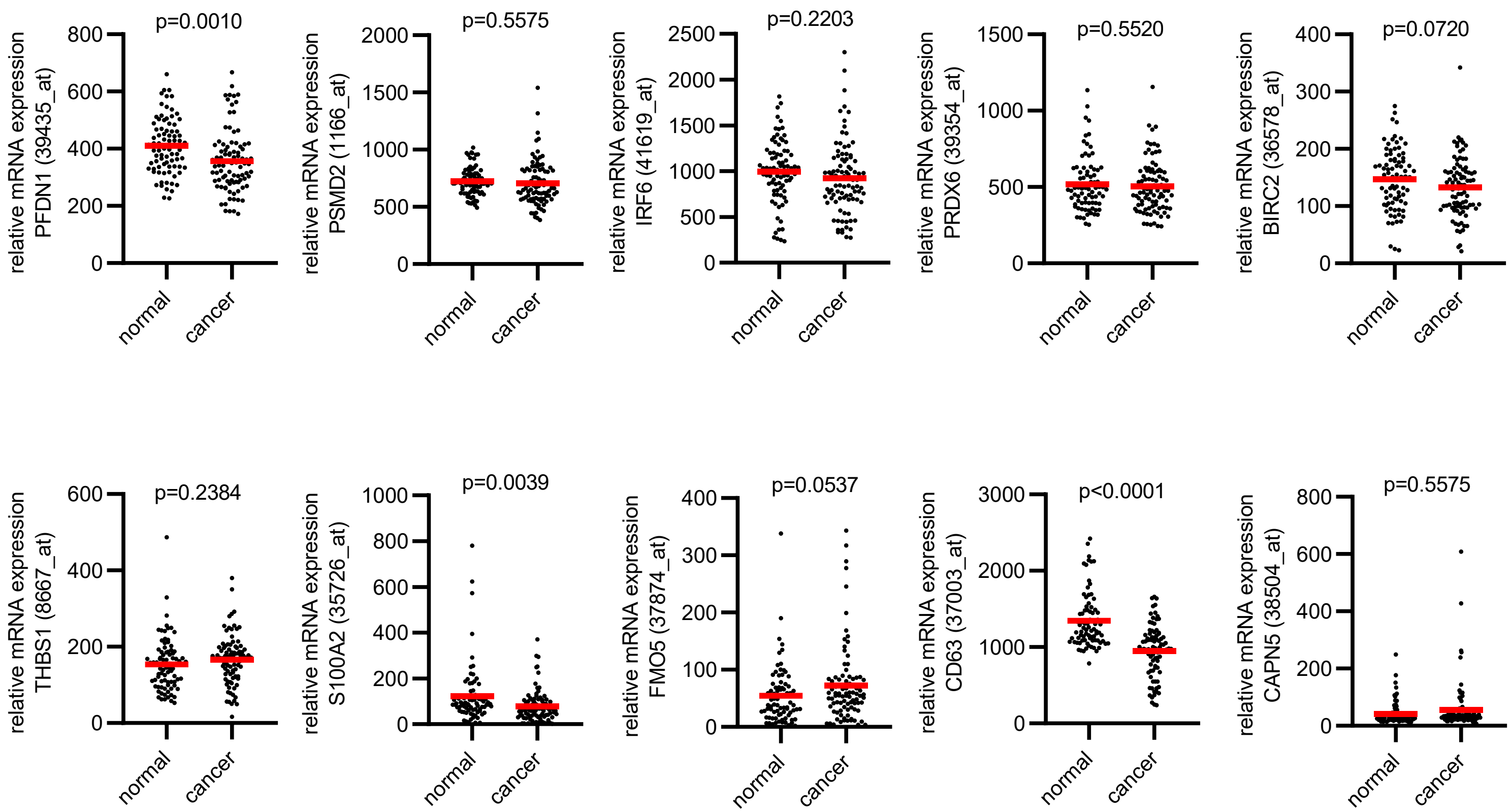

Supplementary Figure S3: *In silico* validation of candidate biomarker transcripts in in dataset GSE70768 (Gene Expression Omnibus). Expression levels of the indicated genes (*PFDN1, PSMD2, IRF6, PRDX6, BIRC2, THBS1, S100A2, CD63, FMO5*, and *CAPN5*) were analyzed using microarray expression data from the GSE70768 dataset (Gene Expression Omnibus). Probe identifiers corresponding to each gene are indicated in parentheses in the figure panels. Relative mRNA expression levels are shown for normal prostate tissue samples (n = 81) and prostate cancer samples (n = 90). Each dot represents an individual patient sample, and the red horizontal line indicates the mean expression level for each group. Differences in gene expression between the normal and cancer groups were evaluated using an unpaired two-tailed Student's t-test, which compares the mean expression levels between the two independent groups. The p-values shown in each panel correspond to this comparison between normal and cancer samples. This analysis represents a single *in silico* evaluation of publicly available transcriptomic data, and each data point corresponds to an independent patient sample. P-values are as indicated in the figure.
