# Supplementary material for: Transcriptional profiling of circulating extracellular vesicles from prebiopsy prostate cancer patients
Source: Mol Oncol. 2026 Mar 26;20(11):2647–55. doi: 10.1002/1878-0261.70244 (PMC13398947; doi:10.1002/1878-0261.70244)
Supplement: Supplementary file 3 [file MOL2-20-2647-s004.pdf]

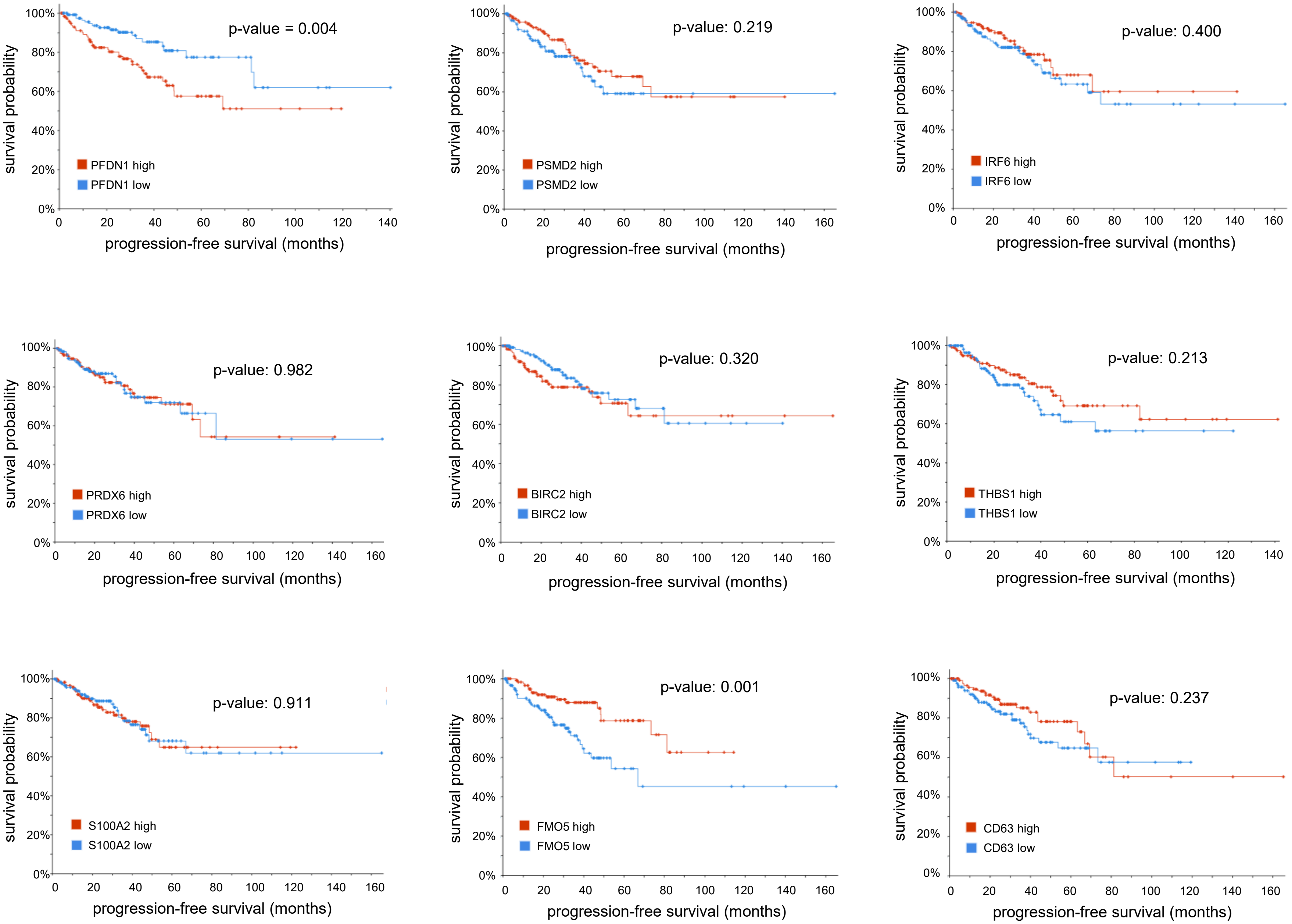

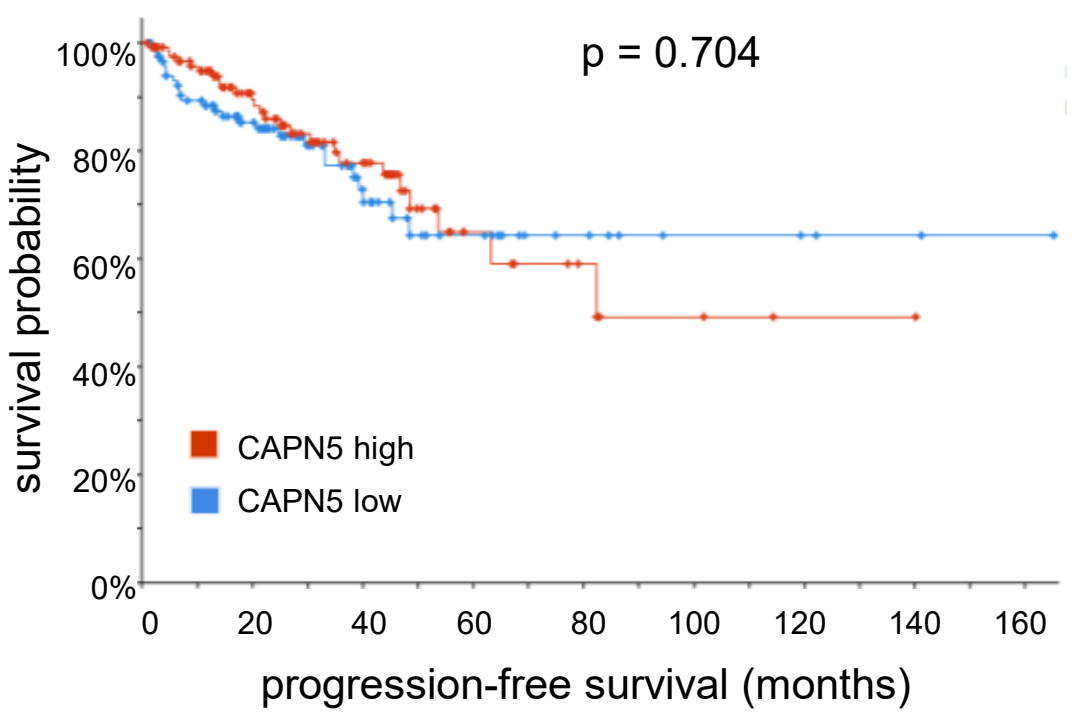

Supplementary Figure S2. Association between candidate biomarker transcript expression and progression-free survival in prostate cancer. Kaplan–Meier survival analysis of progression-free survival (PFS) in prostate adenocarcinoma patients from the TCGA PanCancer dataset according to expression levels of the indicated genes (*PFDN1, PSMD2, IRF6, PRDX6, BIRC2, THBS1, S100A2, FMO5, CD63*, and *CAPN5*). Expression data were derived from RNA-sequencing measurements. Patients were stratified based on gene expression levels, and extreme quartiles were compared, with the highest expression quartile (top 25%, red curves) compared to the lowest expression quartile (bottom 25%, blue curves). A total of n = 486 informative patient cases were included in the survival analysis. Kaplan–Meier curves show the probability of progression-free survival over time (months) for each group. Differences in survival distributions between the high- and low-expression groups were evaluated using the log-rank test, which compares the survival curves across the entire follow-up period. The p-values shown in each panel correspond to the statistical comparison between the high-expression and low-expression patient groups for the indicated gene. This analysis represents a single *in silico* evaluation of publicly available TCGA transcriptomic and clinical data, and each data point reflects an independent patient sample.
