# Supplementary material for: Transcriptional profiling of circulating extracellular vesicles from prebiopsy prostate cancer patients
Source: Mol Oncol. 2026 Mar 26;20(11):2647–55. doi: 10.1002/1878-0261.70244 (PMC13398947; doi:10.1002/1878-0261.70244)
Supplement: Supplementary file 4 [file MOL2-20-2647-s001.docx]

# Supplementary Table S1. Sample and patient characteristics.

## Descriptive statistics of numeric variables

| Statistic | PSA (ng/ml) | AGE |
| --- | --- | --- |
| count | 77.0 | 88.0 |
| mean | 90.64 | 63.49 |
| std | 536.04 | 10.39 |
| min | 1.0 | 0.0 |
| 25% | 4.0 | 59.0 |
| 50% | 7.0 | 64.0 |
| 75% | 15.0 | 70.0 |
| max | 4640.0 | 86.0 |

## Group comparison (noPCa/lowrisk vs. signPCA)

| Variable | Mean (noPCa/lowrisk) | Mean (signPCA) | p-value |
| --- | --- | --- | --- |
| PSA (ng/ml) | 7.161 | 150.089 | 0.177 |
| Age (years) | 64.065 | 64.022 | 0.985 |

## Sample and patient characteristics

| **Sample_ID** | **Sample_Description** | **included** | **Biopsie_Summary_Gleason** | **Strata_one** | **PSA (ng/ml)** | **AGE** |
| --- | --- | --- | --- | --- | --- | --- |
| 1805-A036285 | Exosomes from Plasma | yes | no tumor | noPCa/lowrisk | 22.0 | 59.0 |
| 1805-A036288 | Exosomes from Plasma | yes | 3+3 | noPCa/lowrisk | 4.0 | 66.0 |
| 1805-A036292 | Exosomes from Plasma | yes | no tumor | noPCa/lowrisk | 8.0 | 67.0 |
| 1805-A036303 | Exosomes from Plasma | yes | no tumor | noPCa/lowrisk | 3.0 | 54.0 |
| 1805-A036311 | Exosomes from Plasma | yes | no tumor | noPCa/lowrisk | 6.0 | 56.0 |
| 1805-A036352 | Exosomes from Plasma | yes | healthy donor | noPCa/lowrisk | nan | 58.0 |
| 1805-A036355 | Exosomes from Plasma | yes | healthy donor | noPCa/lowrisk | nan | 58.0 |
| 1806-A036291 | Exosomes from Plasma | yes | no tumor | noPCa/lowrisk | 2.0 | 61.0 |
| 1806-A036293 | Exosomes from Plasma | yes | no tumor | noPCa/lowrisk | 4.0 | 59.0 |
| 1806-A036309 | Exosomes from Plasma | yes | no tumor | noPCa/lowrisk | 15.0 | 71.0 |
| 1806-A036333 | Exosomes from Plasma | yes | no tumor | noPCa/lowrisk | 4.0 | 69.0 |
| 1806-A036334 | Exosomes from Plasma | yes | no tumor | noPCa/lowrisk | 3.0 | 63.0 |
| 1806-A036347 | Exosomes from Plasma | yes | healthy donor | noPCa/lowrisk | nan | 69.0 |
| 1806-A036353 | Exosomes from Plasma | yes | healthy donor | noPCa/lowrisk | nan | 63.0 |
| 1805-A036268 | Exosomes from Plasma | yes | 4+3+5 | signPCA | 4.0 | 67.0 |
| 1805-A036282 | Exosomes from Plasma | yes | 3+4 | signPCA | 14.0 | 70.0 |
| 1805-A036336 | Exosomes from Plasma | yes | 3+4 | signPCA | 5.0 | 55.0 |
| 1806-A036276 | Exosomes from Plasma | yes | 4+3+5 | signPCA | 5.0 | 64.0 |
| 1806-A036295 | Exosomes from Plasma | yes | 3+4 | signPCA | 7.0 | 70.0 |
| 1806-A036300 | Exosomes from Plasma | yes | 3+4 | signPCA | 6.0 | 64.0 |
| 1806-A036301 | Exosomes from Plasma | yes | 3+4 | signPCA | 21.0 | 55.0 |
| 1806-A036310 | Exosomes from Plasma | yes | 4+3 | signPCA | 20.0 | 73.0 |
| 1806-A036323 | Exosomes from Plasma | yes | 3+4 | signPCA | 10.0 | 59.0 |
| 1806-A036324 | Exosomes from Plasma | yes | 3+4 | signPCA | 10.0 | 53.0 |
| 1806-A036331 | Exosomes from Plasma | yes | 3+4 | signPCA | 6.0 | 61.0 |
| 1806-A036332 | Exosomes from Plasma | yes | 3+4 | signPCA | 27.0 | 67.0 |
| 1806-A036339 | Exosomes from Plasma | yes | 3+4+5 | signPCA | 9.0 | 74.0 |
| 1806-A036340 | Exosomes from Plasma | yes | 4+3+5 | signPCA | 16.0 | 53.0 |
| 1805-A036284 | Exosomes from Plasma | no | nan | noPCa/lowrisk | 21.0 | 47.0 |
| 1805-A036287 | Exosomes from Plasma | no | nan | noPCa/lowrisk | 14.0 | 71.0 |
| 1805-A036302 | Exosomes from Plasma | no | 3+3 | noPCa/lowrisk | 7.0 | 66.0 |
| 1805-A036305 | Exosomes from Plasma | no | no tumor | noPCa/lowrisk | 4.0 | 71.0 |
| 1805-A036307 | Exosomes from Plasma | no | no tumor | noPCa/lowrisk | 11.0 | 67.0 |
| 1805-A036313 | Exosomes from Plasma | no | no tumor | noPCa/lowrisk | 1.0 | 48.0 |
| 1805-A036315 | Exosomes from Plasma | no | no tumor | noPCa/lowrisk | 17.0 | 61.0 |
| 1805-A036318 | Exosomes from Plasma | no | no tumor | noPCa/lowrisk | 8.0 | 64.0 |
| 1805-A036319 | Exosomes from Plasma | no | no tumor | noPCa/lowrisk | 9.0 | 73.0 |
| 1805-A036327 | Exosomes from Plasma | no | no tumor | noPCa/lowrisk | 4.0 | 70.0 |
| 1805-A036338 | Exosomes from Plasma | no | no tumor | noPCa/lowrisk | 5.0 | 66.0 |
| 1805-A036342 | Exosomes from Plasma | no | no tumor | noPCa/lowrisk | 4.0 | 75.0 |
| 1805-A036348 | Exosomes from Plasma | no | healthy donor | noPCa/lowrisk | nan | 62.0 |
| 1805-A036349 | Exosomes from Plasma | no | healthy donor | noPCa/lowrisk | nan | 59.0 |
| 1805-A036351 | Exosomes from Plasma | no | healthy donor | noPCa/lowrisk | nan | 70.0 |
| 1805-A036354 | Exosomes from Plasma | no | healthy donor | noPCa/lowrisk | nan | 55.0 |
| 1805-A036356 | Exosomes from Plasma | no | healthy donor | noPCa/lowrisk | nan | 58.0 |
| 1806-A036272 | Exosomes from Plasma | no | 3+3 | noPCa/lowrisk | 4.0 | 60.0 |
| 1806-A036278 | Exosomes from Plasma | no | no tumor | noPCa/lowrisk | 2.0 | 66.0 |
| 1806-A036286 | Exosomes from Plasma | no | no tumor | noPCa/lowrisk | 7.0 | 76.0 |
| 1806-A036294 | Exosomes from Plasma | no | no tumor | noPCa/lowrisk | 4.0 | 57.0 |
| 1806-A036297 | Exosomes from Plasma | no | no tumor | noPCa/lowrisk | 7.0 | 67.0 |
| 1806-A036299 | Exosomes from Plasma | no | 3+3 | noPCa/lowrisk | 5.0 | 64.0 |
| 1806-A036304 | Exosomes from Plasma | no | no tumor | noPCa/lowrisk | 9.0 | 63.0 |
| 1806-A036317 | Exosomes from Plasma | no | no tumor | noPCa/lowrisk | 4.0 | 53.0 |
| 1806-A036337 | Exosomes from Plasma | no | no tumor | noPCa/lowrisk | 4.0 | 76.0 |
| 1806-A036345 | Exosomes from Plasma | no | 3+3 | noPCa/lowrisk | 3.0 | nan |
| 1806-A036350 | Exosomes from Plasma | no | healthy donor | noPCa/lowrisk | nan | 43.0 |
| 1806-A036357 | Exosomes from Plasma | no | healthy donor | noPCa/lowrisk | nan | 63.0 |
| 1806-A036358 | Exosomes from Plasma | no | healthy donor | noPCa/lowrisk | nan | 62.0 |
| 1805-A036267 | Exosomes from Plasma | no | 3+4 | signPCA | 6.0 | 69.0 |
| 1805-A036273 | Exosomes from Plasma | no | 3+4 | signPCA | 8.0 | 70.0 |
| 1805-A036274 | Exosomes from Plasma | no | 3+3 | signPCA | 12.0 | 53.0 |
| 1805-A036275 | Exosomes from Plasma | no | 4+3+5 | signPCA | 41.0 | 71.0 |
| 1805-A036280 | Exosomes from Plasma | no | 3+4 | signPCA | 7.0 | 73.0 |
| 1805-A036281 | Exosomes from Plasma | no | 3+4 | signPCA | 2.0 | 63.0 |
| 1805-A036283 | Exosomes from Plasma | no | 3+3 | signPCA | 16.0 | 60.0 |
| 1805-A036289 | Exosomes from Plasma | no | 3+4 | signPCA | 18.0 | 55.0 |
| 1805-A036290 | Exosomes from Plasma | no | 3+3 | signPCA | 19.0 | 67.0 |
| 1805-A036306 | Exosomes from Plasma | no | 3+4 | signPCA | 20.0 | 66.0 |
| 1805-A036308 | Exosomes from Plasma | no | 4+5+3 | signPCA | 4640.0 | 66.0 |
| 1805-A036321 | Exosomes from Plasma | no | 4+3+5 | signPCA | 197.0 | 76.0 |
| 1805-A036322 | Exosomes from Plasma | no | 3+4+5 | signPCA | 11.0 | 63.0 |
| 1805-A036326 | Exosomes from Plasma | no | 3+4+5 | signPCA | 24.0 | 75.0 |
| 1805-A036329 | Exosomes from Plasma | no | 3+4 | signPCA | 3.0 | 51.0 |
| 1805-A036335 | Exosomes from Plasma | no | 4+5+3 | signPCA | 15.0 | 66.0 |
| 1805-A036341 | Exosomes from Plasma | no | 3+4+5 | signPCA | 7.0 | 60.0 |
| 1805-A036346 | Exosomes from Plasma | no | 3+4+5 | signPCA | 3.0 | 0.0 |
| 1806-A036269 | Exosomes from Plasma | no | 4+3+5 | signPCA | 180.0 | 86.0 |
| 1806-A036270 | Exosomes from Plasma | no | no tumor | signPCA | 2.0 | 69.0 |
| 1806-A036271 | Exosomes from Plasma | no | 3+4 | signPCA | 8.0 | 61.0 |
| 1806-A036277 | Exosomes from Plasma | no | 4+3+5 | signPCA | 11.0 | 69.0 |
| 1806-A036279 | Exosomes from Plasma | no | 3+4 | signPCA | 6.0 | 62.0 |
| 1806-A036296 | Exosomes from Plasma | no | 5+4+3 | signPCA | 3.0 | 70.0 |
| 1806-A036298 | Exosomes from Plasma | no | 3+4+5 | signPCA | 6.0 | 72.0 |
| 1806-A036312 | Exosomes from Plasma | no | 4+3+5 | signPCA | 695.0 | 49.0 |
| 1806-A036314 | Exosomes from Plasma | no | 5+4 | signPCA | 614.0 | 60.0 |
| 1806-A036316 | Exosomes from Plasma | no | 3+4 | signPCA | 4.0 | 79.0 |
| 1806-A036325 | Exosomes from Plasma | no | 3+4 | signPCA | 6.0 | 73.0 |
| 1806-A036330 | Exosomes from Plasma | no | 4+3+5 | signPCA | 6.0 | 70.0 |
| 1806-A036343 | Exosomes from Plasma | no | 3+4 | signPCA | 4.0 | 72.0 |
| 1805-A036360 | Exosomes from Cells* | no | technical validation |  |  |  |
| 1806-A036359 | Exosomes from Cells* | no | technical validation |  |  |  |
| 1806-A036361 | Exosomes from Cells* | no | technical validation |  |  |  |

- These samples were used as technical references only
