# Supplementary material for: Transcriptional profiling of circulating extracellular vesicles from prebiopsy prostate cancer patients
Source: Mol Oncol. 2026 Mar 26:10.1002/1878-0261.70244. Online ahead of print. doi: 10.1002/1878-0261.70244 (PMC13398947; doi:10.1002/1878-0261.70244)
Supplement: Supplementary file 1 — Fig. S1. Expression of candidate biomarker transcripts across prostate cancer tumor stages in the TCGA PanCancer cohort. [file MOL2-9999-0-s002.pdf]

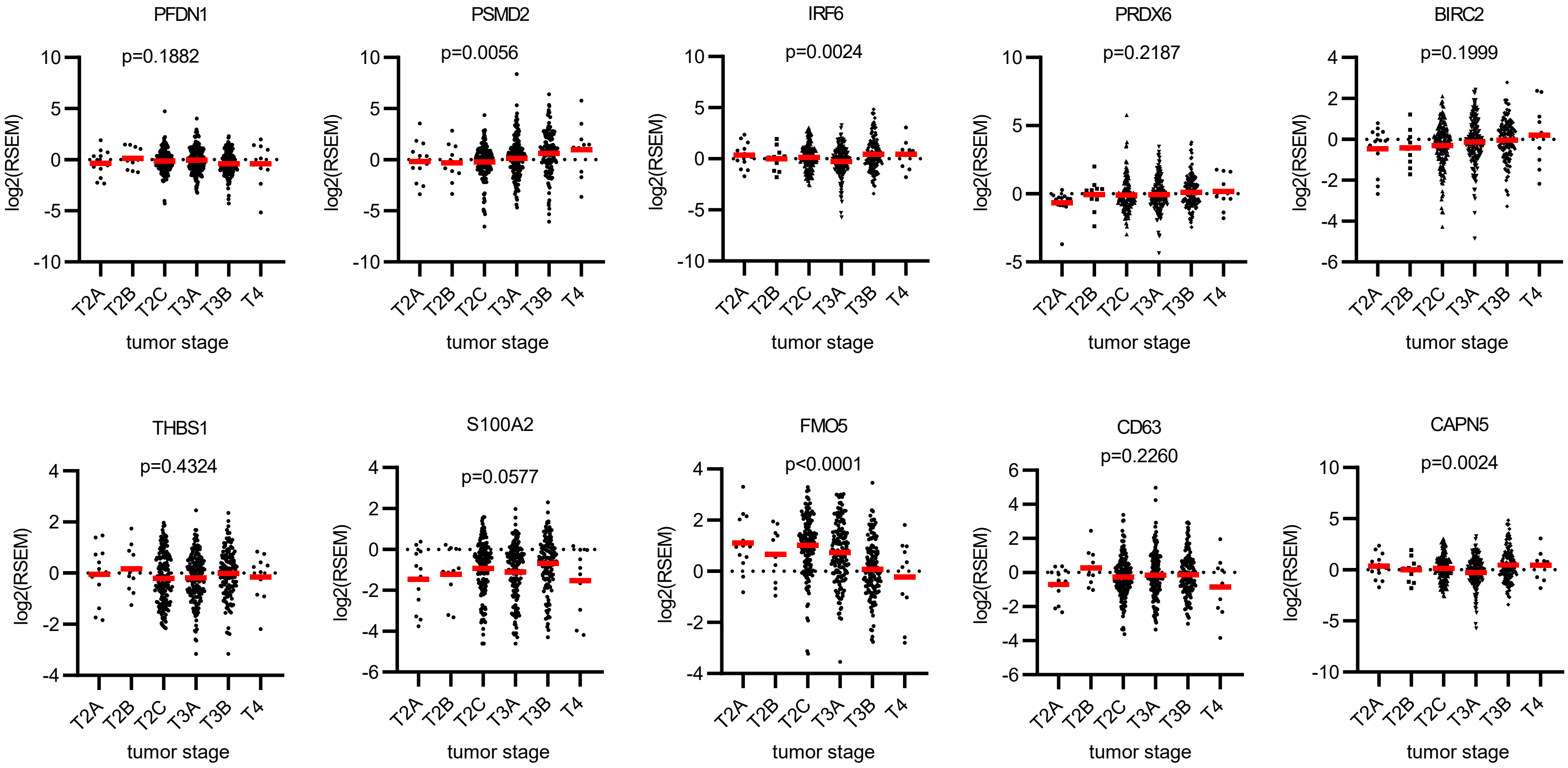

Supplementary Figure S1. Expression of candidate biomarker transcripts across prostate cancer tumor stages in the TCGA PanCancer cohort. Gene expression levels *of PFDN1, PSMD2, IRF6, PRDX6, BIRC2, THBS1, S100A2, FMO5, CD63*, and *CAPN5* were analyzed using RNA-sequencing data from the TCGA PanCancer prostate adenocarcinoma dataset. Expression values are presented as log_2-transformed RSEM-normalized counts for individual tumor samples (black dots). Red horizontal lines indicate the median expression level for each tumor stage group.Tumor stages were classified according to the AJCC tumor staging system, including T2A (n = 13), T2B (n = 10), T2C (n = 163), T3A (n = 157), T3B (n = 133), and T4 (n = 10). Each data point represents an independent patient sample.Differences in gene expression among tumor stages were evaluated using the Kruskal–Wallis nonparametric test, which assesses whether the distribution of gene expression differs across all tumor stage groups simultaneously. The p-values shown in each panel correspond to the overall comparison across the six tumor stage groups, rather than pairwise comparisons between individual stages. This analysis represents a single in silico evaluation of publicly available TCGA transcriptomic data, and each data point corresponds to an individual tumor sample. P values are shown in the figure.
